# Role of Digital Engagement in Diabetes Care Beyond Measurement: Retrospective Cohort Study

**DOI:** 10.2196/24030

**Published:** 2021-02-18

**Authors:** Yifat Fundoiano-Hershcovitz, Abigail Hirsch, Sharon Dar, Eitan Feniger, Pavel Goldstein

**Affiliations:** 1 DarioHealth Caesarea Israel; 2 School of Public Health University of Haifa Haifa Israel

**Keywords:** blood glucose, mHealth, diabetes, self-management, digital engagement

## Abstract

**Background:**

The use of remote data capture for monitoring blood glucose and supporting digital apps is becoming the norm in diabetes care. One common goal of such apps is to increase user awareness and engagement with their day-to-day health-related behaviors (digital engagement) in order to improve diabetes outcomes. However, we lack a deep understanding of the complicated association between digital engagement and diabetes outcomes.

**Objective:**

This study investigated the association between digital engagement (operationalized as tagging of behaviors alongside glucose measurements) and the monthly average blood glucose level in persons with type 2 diabetes during the first year of managing their diabetes with a digital chronic disease management platform. We hypothesize that during the first 6 months, blood glucose levels will drop faster and further in patients with increased digital engagement and that difference in outcomes will persist for the remainder of the year. Finally, we hypothesize that disaggregated between- and within-person variabilities in digital engagement will predict individual-level changes in blood glucose levels.

**Methods:**

This retrospective real-world analysis followed 998 people with type 2 diabetes who regularly tracked their blood glucose levels with the Dario digital therapeutics platform for chronic diseases. Subjects included “nontaggers” (users who rarely or never used app features to notice and track mealtime, food, exercise, mood, and location, n=585) and “taggers” (users who used these features, n=413) representing increased digital engagement. Within- and between-person variabilities in tagging behavior were disaggregated to reveal the association between tagging behavior and blood glucose levels. The associations between an individual’s tagging behavior in a given month and the monthly average blood glucose level in the following month were analyzed for quasicausal effects. A generalized mixed piecewise statistical framework was applied throughout.

**Results:**

Analysis revealed significant improvement in the monthly average blood glucose level during the first 6 months (*t*=−10.01, *P*<.001), which was maintained during the following 6 months (*t*=−1.54, *P*=.12). Moreover, taggers demonstrated a significantly steeper improvement in the initial period relative to nontaggers (*t*=2.15, *P*=.03). Additional findings included a within-user quasicausal nonlinear link between tagging behavior and glucose control improvement with a 1-month lag. More specifically, increased tagging behavior in any given month resulted in a 43% improvement in glucose levels in the next month up to a person-specific average in tagging intensity (*t*=−11.02, *P*<.001). Above that within-person mean level of digital engagement, glucose levels remained stable but did not show additional improvement with increased tagging (*t*=0.82, *P*=.41). When assessed alongside within-person effects, between-person changes in tagging behavior were not associated with changes in monthly average glucose levels (*t*=1.30, *P*=.20).

**Conclusions:**

This study sheds light on the source of the association between user engagement with a diabetes tracking app and the clinical condition, highlighting the importance of within-person changes versus between-person differences. Our findings underscore the need for and provide a basis for a personalized approach to digital health.

## Introduction

Diabetes mellitus is characterized by hyperglycemia that can reduce life expectancy [1], cause considerable health complications, increase cost of care, and lower quality of life [2,3]. The treatment of diabetes mellitus is challenging for both persons with diabetes and clinicians because successful management requires sustained patient-driven lifestyle changes [4,5]. For many, the fundamental challenge of managing chronic diabetes is doing what is needed rather than knowing what to do per se. Research suggests that patients need more than theoretical knowledge about healthy eating, exercise, and self-monitoring of blood glucose [6]. They also need assistance building awareness of their daily health-related behaviors. This awareness building and engagement with prohealth behaviors seeds the implementation of a prohealth lifestyle [7-10].

Technology-driven solutions can help persons with type 2 diabetes bridge the gap between knowing what to do, building awareness and engagement, and implementing these changes [11,12]. Mobile apps have been shown to improve diabetic outcomes via education and support for adhering to evidence-based recommendations [13-16]. Apps for diabetes management and diabetes online communities appear to be useful tools for helping people with type 2 diabetes to control HbA_1c_ and are increasingly considered core intervention tools in self-management for patients with type 2 diabetes [17-19].

Such apps often include the following two core features: a method for recording blood glucose measurements and a vehicle for logging behaviors and situations that impact health outcomes. Paper-and-pencil logging of activities, such as meals, food intake, and exercise, alongside blood glucose measurements has been a long-standing best practice for building awareness and helping individuals better control their glucose levels. In the emerging world of digital diabetes care, tagging (creating a digital in-app activity log) represents a convenient alternative for activity tracking that can be leveraged for app-based diabetes self-management [20].

Health behavior change theory posits that new health behaviors emerge when people gain both knowledge and self-efficacy to implement the said knowledge [21-23]. We posit that the moment of marking (tagging) one’s context in conjunction with taking a blood glucose measurement is a prime opportunity for reinforcing knowledge and building self-efficacy. It is possible that what is being tagged is of less importance than the act of tagging something. In other words, by tagging with measurement, persons with type 2 diabetes transform each glucose reading into a moment of quick reflection on their context and actions proceeding that measurement. This moment of focused awareness building may be a key piece in launching a virtuous process of improved future health behavior.

However, as the usage of apps to capture blood glucose data and to log behavior increases, sophisticated analysis of the rich data now available has lagged. Research gaps include understanding the general blood glucose trajectory among persons with type 2 diabetes using digital diabetes support tool users, the association between app engagement and short- and long-term clinical outcomes, and the relative impact of specific app features dedicated to self-management [11,15,24]. In addition, strikingly little work has focused on disentangling the value of remote digital capture of glucose measurements versus digital engagement via tagging. Nuanced modeling of the impact of different features within diabetes apps could help to maximize the impact of mobile health apps on behavior change and, by extension, on health outcomes [25]. Of note, previous studies suggested that changes in diabetes clinical outcomes appear to have the following two phases: an initial improvement over 6 months, followed by a longer-term sustained period [26,27]. Modeling that allows for a multitrajectory process, that is, for change trajectories to have different slopes at different periods of time, while not the norm in many assessments of digital health platforms, seems imperative.

Over the last decade, behavioral science research has increasingly focused on between-person processes as opposed to within-person processes [28]. Surprisingly, the quantitative literature on diabetes still generally emphasizes treatment efficacy and associated between-person group-level factors and ignores within-person variability [29-31]. However, disaggregating between-person and within-person variability can illuminate the dynamics of the relative contribution of intraperson changes versus between-person differences to successful diabetes management. Moreover, this kind of analysis enables testing quasicausal relationships by adding lagged effects between modeled within-person digital engagement and clinical outcomes. Finally, as described above, the associations between digital engagement and clinical outcomes are not necessarily linear, as has been mostly assumed previously [32].

This study leverages a retrospective analysis of a home-use diabetes glucometer with full data capture in a supporting mobile app among type 2 diabetes patients with poorly controlled blood glucose levels. We hypothesized that during the first 6 months of using a chronic condition self-management app, tagging alongside blood glucose measuring would be associated with reduced blood glucose levels. By modeling the two-stage trajectory process, we expected to show the improvement to persist until the end of the 1-year study period. We also hypothesized that disaggregated within- and between-person variabilities in engagement behaviors would be predictive of reductions in monthly average blood glucose levels. Moreover, we suspected that 1-month lagged within-person digital engagement would be associated with improvements in monthly average blood glucose levels.

## Methods

### Platform

**Figure 1:**
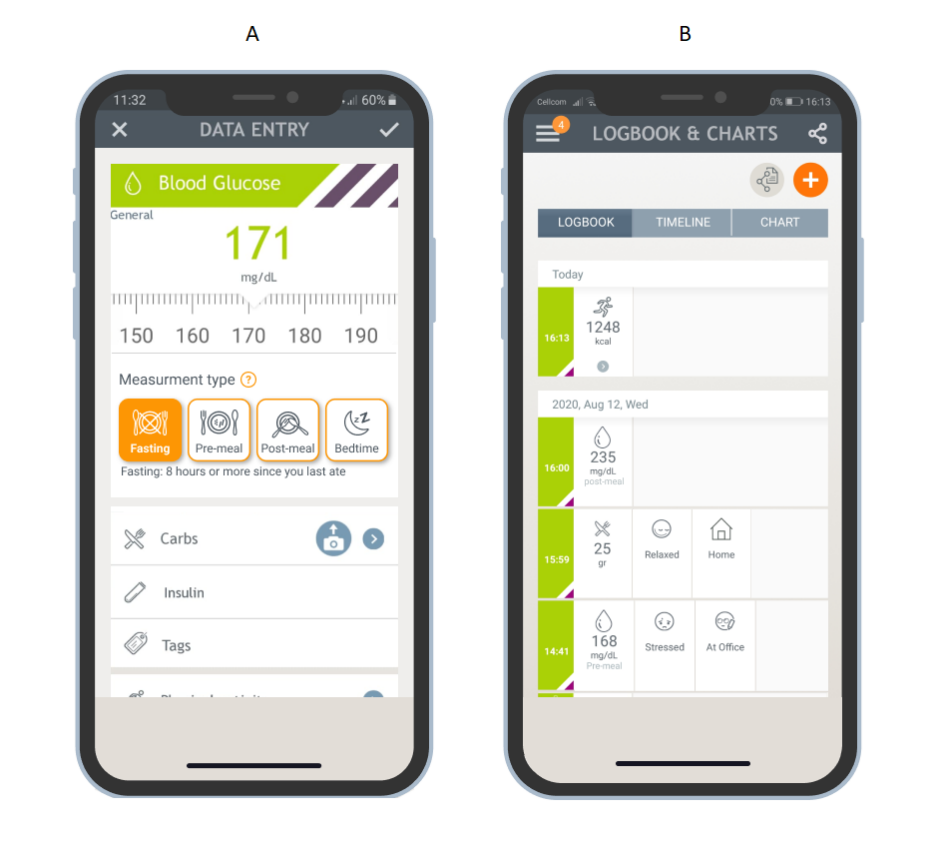
Dario mobile app platform. (A) Data entry screen allows tagging measurement type, carbohydrate intake (grams), physical activity (kcal), and tags such as mood setting and location. (B) Logbook screen presenting measurements and tagging records.

This study utilized the Dario digital therapeutics solution for chronic diseases to support self-management of diabetes. The Dario platform combines an innovative meter with a phone app that is available for both Android and iOS devices. The glucometer consists of a small pocket-sized holder for strips, a lancet, and the meter. The meter is removed from the holder and plugged directly into a cell phone, effectively converting the cell phone into the display screen for the meter. Connecting the meter directly to the phone has two advantages. First, it ensures 100% data capture during glucose readings. Second, it means users have opened the mobile app with each glucose measurement. This makes contextually tagging a measurement very easy to do at the time of taking the measurement. More specifically, the glucose meter is physically attached to the mobile phone, and the measurement is shown on the mobile phone (the meter does not have a screen) in a “decision support system” view. After the measurement is shown, a data entry screen is presented, where additional information can be added. The additional information includes measurement time (fasting/premeal/postmeal/bedtime); carbohydrate intake (grams); meal, mood, and location settings; and physical activity (kcal). All information is stored in the patient log book in the app “attached” to the specific blood glucose reading. Data are uploaded to the cloud for backup and further analysis, as presented in Figure 1. An extended version of this figure is provided in Multimedia Appendix 1.

### Measures

The monthly average blood glucose level, which was defined as the mean of all of a user’s blood glucose measurements taken over a 30-day interval, was used as the core outcome metric. Independent variables included digital engagement, operationalized as the number of times a user added a tag to a measurement each month, and available demographic variables of gender and age. All data were transferred and stored in compliance with Health Insurance Portability and Accountability Act (HIPAA) requirements, using Amazon AWS database services. All data were anonymized before extraction for this study.

### Users

The 998 users included in this analysis used the Dario platform between 2016 and 2020. The inclusion criteria were as follows: type 2 diabetes, noninsulin treatment, first month blood glucose average >180 mg/dL, blood glucose measurements during the first 2 months on the system, and at least five blood glucose measurements during the first and 12th months on the platform.

Users were grouped by their use of the behavioral tagging features of the app. The “taggers” group included users with an average of more than one tag per month over the 12-month activity (n=413). Users who only used the app for blood glucose measurements were designated as “nontaggers” with an average of one or less than one tag per month over the 12-month activity (n=585).

No difference between the groups was found for gender (χ^2^_1_=0.19, *P*=.66), age (*B*=0.96, t_596_=1.20, *P*=.23), initial blood glucose level (*B*=5.89, t_596_=1.64, *P*=.10), and the average number of monthly blood glucose measurements over the study period (*B*=−0.26, t_595_=−0.18, *P*=.85). 

Ethical & Independent Review Services [33], a professional review board, issued the institutional review board exemption for this study (18032-03#).

### Analytical Approach

Statistical analysis was conducted in two stages. The first stage modeled differences in the monthly average blood glucose level throughout users’ initial 12 months on the Dario platform, grouped by taggers and nontaggers. The second analysis focused on the association between disaggregated within- and between-patient tagging behaviors and the monthly average blood glucose level. The test was two-tailed.

#### First Analysis: Testing Differences in the Monthly Average Blood Glucose Level Throughout the Initial 12 Months by Taggers and Nontaggers 

The standard linear longitudinal model assumes a single slope growth pattern for changes in an outcome variable across time. Sometimes, such a simple model does not fit the empirical data. In contrast, piecewise‐based mixed‐effects models allow flexibility in the modeling of variable change trajectories across time [34]. Here, a mixed piecewise model assessed differences in the monthly average blood glucose level in two segments (1-6 months and 7-12 months) with users grouped as taggers and nontaggers. The piecewise model allowed the data to exhibit different linear trends over their different regions. This statistical approach provided an opportunity to model curvilinear changes in the monthly average blood glucose level as a single process and to test complex effects based on this more flexible model. Based on previous research [26], the piecewise cutoff point for the model slopes was chosen at 6 months, assuming a change in the time-related monthly average blood glucose trajectory after 6 months of Dario device usage. We tested several residual distributions of the model outcome (Gaussian, log normal, and gamma) and different combinations of random effects. The model with the best fit, and thus used in the analysis, was based on log‐normal residuals, and it included person-based random intercepts and random slopes for both periods (1-6 months and 7-12 months). The model also included an interaction between the groups (taggers and nontaggers) at both periods.

#### Second Analysis: Assessing Within-Person and Between-Person Associations Between Tagging Behavior and the Monthly Average Blood Glucose Level

The second analysis was performed on the entire sample of users (n=998), with a focus on continuous behavioral tagging within individuals as opposed to trends over time by groups in the first analysis. The monthly overall tagging volume was disaggregated to separate within- and between-person variabilities using person-level centering and person-level aggregation [29]. In addition, 1-month lagged tagging engagement was calculated based on the within-person engagement. Thereafter, a generalized mixed model assuming log-normal outcome residual distribution was applied to test the association of monthly within-person engagement and between-person engagement with the monthly average blood glucose level. The model also included 1-month lagged within-person engagement to test for a quasicausal relationship between a user’s tagging engagement and the monthly average blood glucose level. Since lagged engagement demonstrated a nonlinear relationship with the monthly average blood glucose level, a quadratic term for lagged engagement was also added to the model. 

Finally, we tested a curve-linear pattern of the association between lagged within-person engagement and the monthly average blood glucose level by applying a piecewise generalized mixed model defining two slopes for the relationship with a cutoff point in the person-level mean of the lagged engagement.

## Results

### First Analysis: Piecewise Generalized Mixed Model Analysis

Patients’ age (*B*=0.001, *t*=.87, *P*=.38) and gender (*B*=−0.02, *t*=−1.61, *P*=.11) were not related to the monthly average blood glucose level.

**Figure 2:**
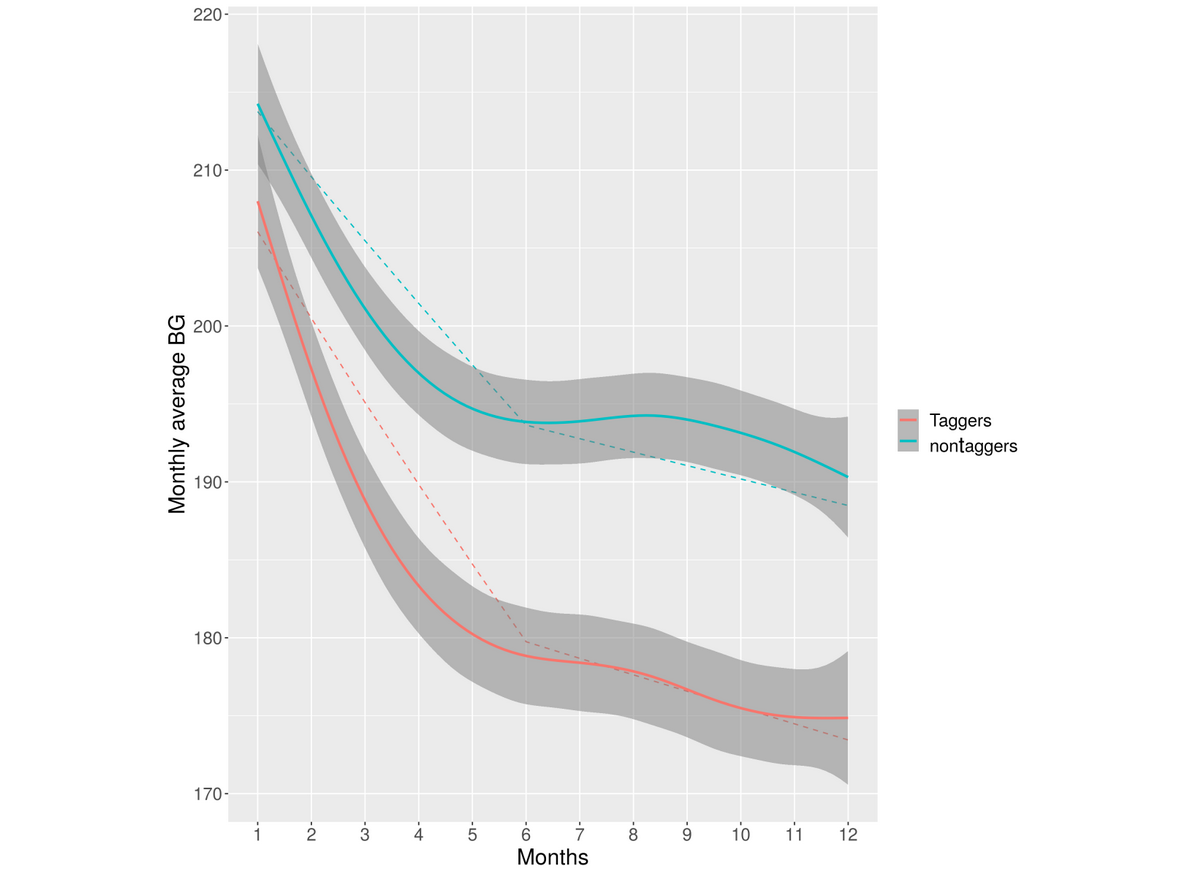
Differences in time-related monthly average blood glucose (BG) (mg/dL) trajectories between taggers and nontaggers. The figure presents locally weighted smoothed monthly average blood glucose data with 95% confidence intervals (the dark grey area surrounding each curve) and predictions based on a generalized mixed piecewise model for taggers (red) and nontaggers (blue).

Piecewise mixed model analysis revealed a significant monthly average blood glucose decrease for both taggers (*B*=−0.027, 95% CI −0.033 to −0.022; monthly average blood glucose decrease=13%) and nontaggers (*B*=−0.020, 95% CI −0.024 to −0.015; monthly average blood glucose decrease=9%) during the period of the first 6 months of use (Figure 2). In addition, the monthly average blood glucose level showed significantly better improvement among taggers than among nontaggers (*B*=0.008, 95% CI 0.001 to 0.014; *t*=2.15, *P*=.03). Extended information is provided in Multimedia Appendix 2. During the period from 7 to 12 months, there were no significant time-related trending monthly average blood glucose levels among taggers (*B*=−0.005, 95% CI −0.014 to 0.001; monthly average blood glucose decrease=3%) and nontaggers (*B*=−0.004, 95% CI −0.011 to 0.002; monthly average blood glucose decrease=2%). Taggers and nontaggers likewise did not show significant differences in their time-related monthly average blood glucose trend (*B*=0.001, 95% CI −0.008 to 0.011; *t*=0.29, *P*=.77) during the second time period (7-12 months).

### Second Analysis: Within- and Between-Person Associations Between Tagging and Health Conditions

**Figure 3:**
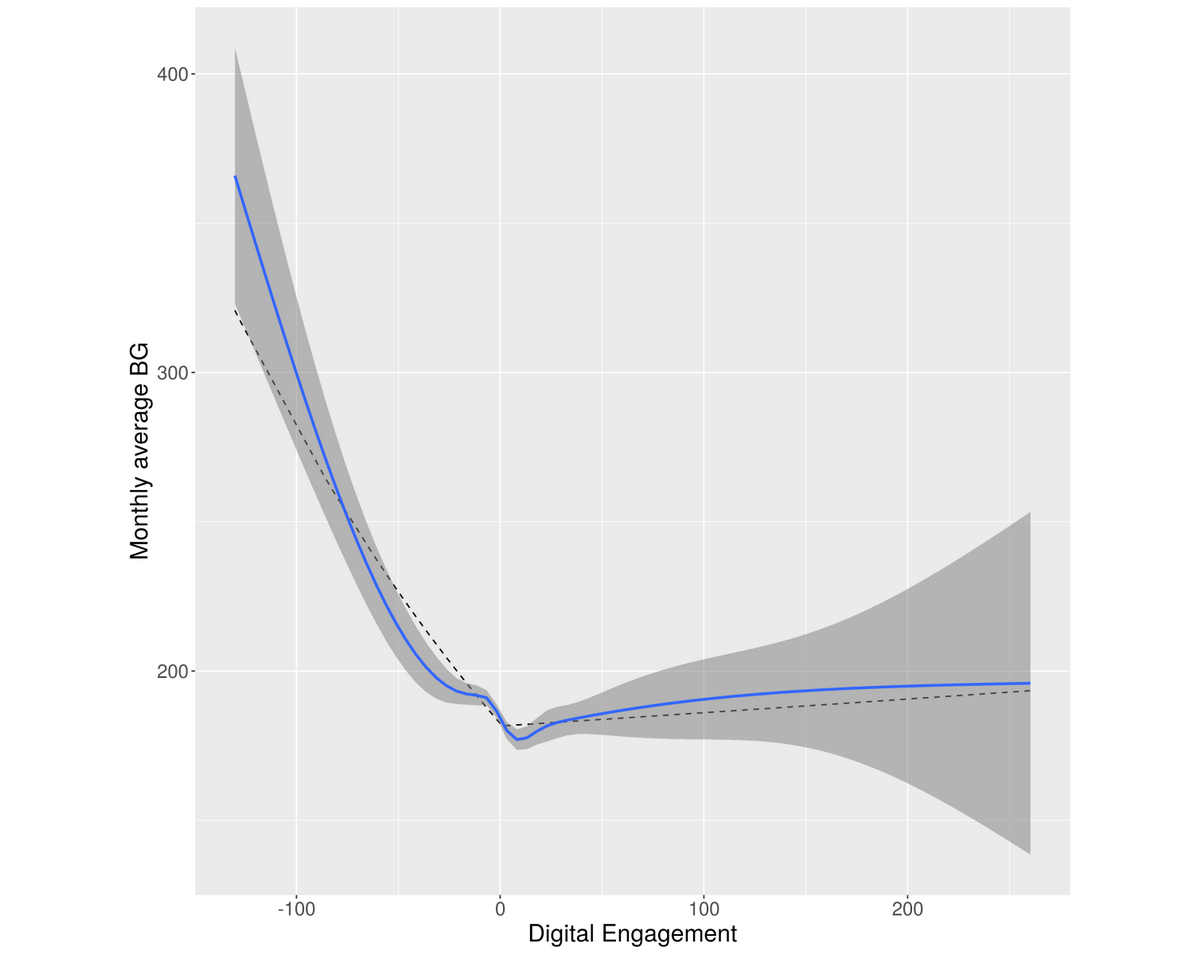
Association between within-person 1-month lagged digital engagement and monthly average blood glucose (BG) (mg/dL). The blue line shows locally weighted smoothing with a 95% confidence interval (the surrounding dark grey area). The dotted gray line indicates results from the generalized mixed piecewise model with two slopes (below and above the person-level mean).

The second analysis focused on the relationship between tagging behaviors and blood glucose levels, decoupling between- and within-person effects as opposed to trends over time examined in the first model. Within-person change in tagging activity was negatively associated with the monthly average blood glucose level (*B*=−0.002, 95% CI −0.0023 to −0.016; *t*=−2.15, *P*=.03) (Figure 3). Extended information is provided in Multimedia Appendix 3. Moreover, preceding month tagging showed a quadratic relationship with the monthly average blood glucose level. Finally, aggregated (between-subject) digital engagement was not related to the monthly average blood glucose level (*B*=0.0005, 95% CI −0.0003 to 0.0012; *t*=1.30, *P*=20). 

For a better understanding of the nonlinear effect that was found between preceding month digital engagement and the absolute monthly average blood glucose level, a piecewise generalized mixed framework was adopted for modeling two slopes of the relationship (below the person-level engagement mean and above the mean) (Figure 3). Up to the subject-level mean, preceding month digital engagement showed a negative association with the monthly average blood glucose level, resulting in a 43% monthly average blood glucose decrease (*B*=−0.004, 95% CI −0.005 to −0.003; *t*=−11.02, *P*<.001). Above the subject-level mean, preceding month digital engagement was not related to the monthly average blood glucose level, showing stable and low monthly average blood glucose levels (*B*=0.0002, 95% CI −0.0003 to 0.0008; *t*=0.82, *P*=.41). 

To better understand the contribution of the single component of digital engagement to the association with blood glucose, we reran the model described above and included measurement time tagging (fasting/premeal/postmeal/bedtime); carbohydrate intake tagging (grams); meal, mood, and location settings; and physical activity tagging (kcal) instead of aggregated tagging. Based on the model, up to the subject-level mean, preceding month carbohydrate intake; meal time tagging; and meal, mood, and location settings showed negative associations with the monthly average blood glucose level (*B*=−0.004, *t*=−3.47, *P*<.001; *B*=−0.007, *t*=−5.56, *P*<.001; and *B*=−0.004, *t*=−6.29, *P*<.001, respectively). Above the subject-level mean, preceding month carbohydrate intake; meal time tagging; and meal, mood, and location settings were not related to the monthly average blood glucose level (*B*=0.002, *t*=1.53, *P*=.13; *B*=−0.0001, *t*=−0.14, *P*=.89; and *B*=−0.0001, *t*=−0.14, *P*=.89, respectively). 

Physical activity tagging showed a similar result pattern but did not reach statistical significance (up to the subject-level mean: *B*=−0.001, *t*=−1.07, *P=*.28; above the subject-level mean: *B*=0.004, *t*=0.08, *P*=.93).

## Discussion

### Principal Results

This real-world analysis presents data analyzing associations between blood glucose levels and digital engagement (tagging) in a digital app for chronic health condition management. More specifically, the results indicate that two distinct phases exist for remote blood glucose monitoring via an app (a rapid improvement phase lasting about 6 months and then a maintenance phase, which was here followed to 12 months). Moreover, the improvement is stronger for users with increased tagging behavior. In addition, disaggregating within- and between-person variabilities in digital engagement, we demonstrated the quasicausal relationship between within-person behavioral tagging in any given month and the blood glucose level in the following month.

Consistent with the literature, we found that users of a connected glucose monitor experienced the most change in their first few months of use [14,15,27]. Of note, change patterns with an early rapid change period followed by a long-tailed period where change is retained appeared in many real-world digital interventions for behavior change [35,36]. While findings of a pre-post intervention change that remains stable after intervention are expected in traditional structured time-bound interventions, most digital health interventions are continuous in nature and thus might be believed to follow a smoother trajectory [37]. Nonetheless, evidence is emerging that there is a distinctly different impact in the short term versus the longer term, even for continuous eHealth interventions. This study shows that utilization of a piecewise mixed model statistical framework appears to be the more appropriate base model to describe a user’s two-phase slope change in blood glucose levels. Likewise, utilization of a piecewise approach allows independent analysis of predictors and covariates for the adoption versus longer-term periods. The piecewise-based model indicates that during the short-term adoption phase, while both taggers and nontaggers show declines in average blood glucose levels, taggers show significantly steeper declines than nontaggers. In other words, tagging appears to build behavioral awareness to life management, contributing to the glucose balance [38]. However, in the longer term, at 7 to 12 months, both groups evidenced flat trajectories, suggesting that over the long term, gains are sustained and durable but not increasing. Building behavioral awareness by means of a digital therapeutics platform addresses barriers to diabetes self-care in the context of everyday life. Previous studies revealed that behavior engagement is associated with increased individual diabetes-related problem-solving ability and with significant improvement in glucose control. Similar to our findings, these improvements were sustained at long-term follow-ups [37,39]. Indeed, following 12 months, the improved glucose level in the taggers group persisted and remained lower than that in the nontaggers group.

Another distinct feature of digital therapeutics is the potential to deliver highly person-centric care. Personalized medicine has been called the “new mantra” in health care [40]. Here too, a move beyond the standard between-subject statistical approach is called for. Disaggregating within- and between-person variabilities in digital engagement enabled evaluation of the association between digital engagement and the monthly average blood glucose level, and in fact, only the within-person component had a significant contribution in predicting the blood glucose level in this model.

Moreover, we demonstrated the quasicausal relationship between within-person behavioral tagging in any given month and the blood glucose level in the following month by applying a piecewise-based mixed model owing to the nonlinear nature of this association. We found a significant lagged association between digital engagement and the monthly average blood glucose level. Increased digital engagement was related to better clinical outcomes when digital engagement was below the person-level average (up to 43% improvement). However, above the person-level average, no association was observed. Here, between-person behavior engagement had no association with the monthly average blood glucose level. In other words, the within-subject component, as opposed to the between-subject component, is the source of the relationship between digital engagement and the blood glucose level.

Recent reviews call for research that moves beyond looking at “do digital health applications work” to more nuanced investigations that disentangle the relative contributions of active ingredients in digital health management protocols [13]. Our findings indicate that the strongest lever for helping people to lower their blood glucose levels is to ensure that they tag each month at least to the level of their personal critical tagging inflection point. Based on these findings, it turns out that just simple boosting of digital engagement to the maximum is not an efficient way to optimize glucose levels in diabetes patients. However, tracking digital engagement for persons with type 2 diabetes and maintaining it just around their average may result in optimal levels of glucose and reduction in patient efforts and digital fatigue. We expect that the analytical approach applied in this study will be beneficial for personalizing interventions and optimizing incentivization planning.

This information could be used to further personalize outreach and incentivization efforts to encourage users to maintain their personal critical level of tagging. At the same time, tagging above the personal mean yields no additional benefit in terms of current or future monthly average blood glucose levels. In other words, messaging that pushes for more tagging is unlikely to drive better glucose levels.

### Limitations

We note several limitations in this study. First, as in all studies involving retrospective real-world data, groups were not randomly assigned and treatment protocols were not prescribed. Both factors create challenges for drawing causal effects. It certainly is possible that people who chose to tag behaviors were those who were the most motivated to change. Our inclusion criteria were designed to ensure that both taggers and nontaggers showed evidence of being motivated about their diabetes care. Fingerstick for regular blood glucose measurement certainly has a higher demand on time and energy than adding a few behavioral tags. All people included in this study were performing measurements regularly over the 12-month period of the study, and there were no differences between groups in terms of the volume of measurements. This would suggest that motivation may not be the primary difference between taggers and nontaggers. At the same time, this also limits the extendibility of the findings to low-measuring and thus presumably low-motivation populations. That said, the within-person analysis of lagged association covers the pitfalls of the classical between-group design, focusing on intrapersonal changes and allowing a quasicausal inference.

In this real-world data analysis, the time scale was designed to reflect monthly interval change over a 12-month period. However, the relationships of interest in this study could be potentially investigated in different scales emphasizing daily, weekly, or monthly fluctuations. Owing to the difficulty in tracking daily changes in digital engagement in real-world studies, most studies focus on monthly fluctuations. Investigating fine-grained measurements with microintervals for tagging would certainly contribute to the literature [31].

Another challenge regarding our data was that available demographic data were limited. While there were no between-group differences by age or gender and no impacts of age and gender on the models, uncontrolled demographic biases might have been present from these or other demographic factors.

### Conclusions

It appears highly likely that tagging features in a chronic condition management app, which are presented at the time of measurement, will help users with type 2 diabetes pause and pay attention to their daily life behaviors and connect these to their blood glucose measurements. Focusing on behavior and context as an integrated part of the glucose measurement process nearly doubled the clinical impact observed in users who only measured blood glucose. Likewise, while there was considerable variability in the volume of tagging, the more a user tagged in a given month, the lower the blood glucose level was likely to be in the next month until a user-specific threshold. Above that threshold, more tagging was not associated with a better clinical outcome.

From a behavioral science perspective, perhaps this is not so surprising. Directing focus onto actionable areas for improvement is likely to queue increased thought and action, and at the same time, the amount of attention to actionable areas needed is likely to vary considerably within individuals.

Future work investigating strategies beyond tagging that drive focus on and execution of actionable prohealth behaviors in a highly personalized within-person manner is certainly needed. Furthermore, similar studies examining piecemeal trajectories and within- versus between-person impacts of other behavior change tactics, including health coaching, gamification, and targeted tips, are warranted. Such a body of literature would help to move the field beyond the current state of “do digital tools work” to a nuanced understanding of what tools drive what clinical outcomes for which people under what circumstances.

## Appendix

Multimedia Appendix 1 Dario mobile app platform. Data entry screen allows tagging measurement time (fasting, premeal, postmeal, and bedtime); carbohydrate intake (grams); meal, mood, and location settings; and physical activity (kcal).

Multimedia Appendix 2 Generalized piecewise mixed model for testing the differences in time-related monthly average blood glucose trajectories between taggers and nontaggers.

Multimedia Appendix 3 Generalized piecewise mixed model for testing the association of within- and between-person engagement with the monthly average blood glucose level.
