# Supplementary figures and images for: Role of Digital Engagement in Diabetes Care Beyond Measurement: Retrospective Cohort Study
Source: JMIR Diabetes. 2021 Feb 18;6(1):e24030. doi: 10.2196/24030 (PMC7932839; doi:10.2196/24030)

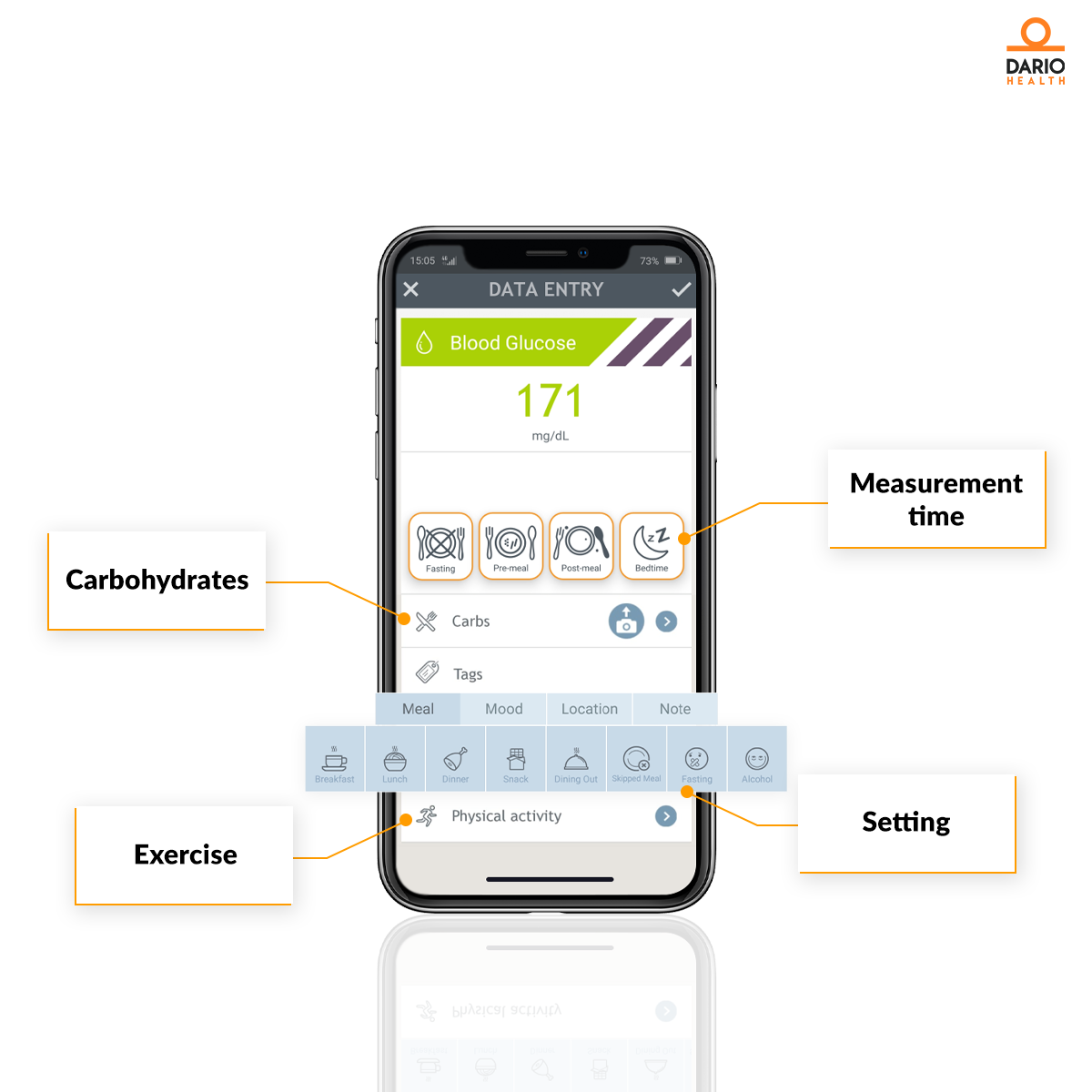

Supplement: Multimedia Appendix 1 [file diabetes_v6i1e24030_app1.png]
