# Supplementary material for: Role of Digital Engagement in Diabetes Care Beyond Measurement: Retrospective Cohort Study
Source: JMIR Diabetes. 2021 Feb 18;6(1):e24030. doi: 10.2196/24030 (PMC7932839; doi:10.2196/24030)
Supplement: Multimedia Appendix 2 [file diabetes_v6i1e24030_app2.docx]

**Multimedia Appendix 2.** Generalized piecewise mixed model for testing the differences in time-related monthly average blood glucose trajectories between taggers and nontaggers.

|  | Average blood glucose | | | |
| --- | --- | --- | --- | --- |
| Predictors | Estimates | CI 95% | T statistic | *P* |
| (Intercept) | 5.36 | 5.32 – 5.40 | 272.60 | <.001 |
| Month1 to 6 | -.03 | -.03 – -.02 | -10.01 | <.001 |
| Month7 to 12 | -.01 | -.01 –.00 | -1.54 | .123 |
| group [non-taggers] | .03 | -.02 –.08 | 1.08 | .281 |
| Month 1 to 6 x group | .01 | .00 –.01 | 2.15 | .032 |
| Month 7 to 12 x group | .00 | -.01 –.01 | .29 | .773 |
| Random Effects* | | | | |
| σ^2^ | 874.98 | | | |
| τ_00_ user.id | 133.48 | | | |
| τ_11_month1 to 6\|user.id. | 1.80 | | | |
| τ_12_ month7 to 12\|user.id. | 4.86 | | | |

* σ^2^ – represents model residuals, τ_00_ , τ_11_ and τ_12_ are random intercept, and random slopes for the time trajectories during 1-6 months and 7-12 months correspondingly.
