# Supplementary material for: Role of Digital Engagement in Diabetes Care Beyond Measurement: Retrospective Cohort Study
Source: JMIR Diabetes. 2021 Feb 18;6(1):e24030. doi: 10.2196/24030 (PMC7932839; doi:10.2196/24030)
Supplement: Multimedia Appendix 3 [file diabetes_v6i1e24030_app3.docx]

**Multimedia Appendix 3.** Generalized piecewise mixed model for testing the association of within- and between-person engagement with the monthly average blood glucose level.

|  | Monthly average blood glucose | | | |
| --- | --- | --- | --- | --- |
| Predictors | Estimates | CI | T statistic | *P* |
| (Intercept) | 5.3853 | 5.3652 – 5.4054 | 524.9186 | <.001 |
| Month1 to 6 | -.0378 | -.1993 –.1238 | -.4582 | .647 |
| Month 7 to 12 | -.0009 | -.1720 –.1703 | -.0099 | .992 |
| Digital engagement (centered) | -.0020 | -.0024 – -.0017 | -10.9896 | <.001 |
| Digital Engagement (centered +lagged) | -.0009 | -.0012 – -.0005 | -4.8818 | <.001 |
| Digital engagement (centered +lagged)^2^ | .0007 | .0004 –.0010 | 4.9265 | <.001 |
| Digital engagement (aggregated) | .0005 | -.0003 –.0012 | 1.2958 | 0.195 |
| Random Effects * | | | | |
| σ^2^ | 724.02 | | | |
| τ_00_ _user.id_ | 0.00 | | | |
| τ_11. Month1 to 6\|_ _user.id_ | 2.80 | | | |
| τ_12_ _Month 7 to 12 \|_ _user.id_ | 3.15 | | | |
|  |  | | | |

* σ^2^ – represents model residuals, τ_00_ , τ_11_ and τ_12_ are random intercept, and random slopes for the time trajectories during 1-6 months and 7-12 months correspondingly.
